# Plant single-cell solutions for energy and the environment

**DOI:** 10.1038/s42003-021-02477-4

**Published:** 2021-08-12

**Authors:** Benjamin Cole, Dominique Bergmann, Crysten E. Blaby-Haas, Ian K. Blaby, Kristofer E. Bouchard, Siobhan M. Brady, Doina Ciobanu, Devin Coleman-Derr, Samuel Leiboff, Jenny C. Mortimer, Tatsuya Nobori, Seung Y. Rhee, Jeremy Schmutz, Blake A. Simmons, Anup K. Singh, Neelima Sinha, John P. Vogel, Ronan C. O’Malley, Axel Visel, Diane E. Dickel

**Affiliations:** 1 DOE Joint Genome Institute, Lawrence Berkeley National Laboratory, Berkeley, CA USA; 2 Department of Biology, Stanford University and Howard Hughes Medical Institute, Stanford, CA USA; 3 Biology Department, Brookhaven National Laboratory, Upton, NY USA; 4 Department of Biochemistry and Cell Biology, Stony Brook University, Stony Brook, NY USA; 5 Environmental Genomics and Systems Biology Division, Lawrence Berkeley National Laboratory, Berkeley, CA USA; 6 Biological Systems and Engineering Division, Lawrence Berkeley National Laboratory, Berkeley, CA USA; 7 Computational Research Division, Lawrence Berkeley National Laboratory, Berkeley, CA USA; 8 Helen Wills Neuroscience Institute & Redwood Center for Theoretical Neuroscience, University of California, Berkeley, CA USA; 9 Department of Plant Biology, University of California, Davis, CA USA; 10 Genome Center, University of California, Davis, CA USA; 11 Plant Gene Expression Center, USDA ARS, Albany, CA USA; 12 Department of Botany and Plant Pathology, Oregon State University, Corvallis, OR USA; 13 Joint BioEnergy Institute, Emeryville, CA USA; 14 School of Agriculture, Food and Wine, Waite Research Institute, Waite Research Precinct, University of Adelaide, Glen Osmond, SA Australia; 15 Plant Biology Laboratory and Howard Hughes Medical Institute, Salk Institute for Biological Studies, La Jolla, CA USA; 16 Carnegie Institution for Science, Department of Plant Biology, Stanford, CA USA; 17 HudsonAlpha Institute for Biotechnology, Huntsville, AL USA; 18 Sandia National Laboratories, Livermore, CA USA; 19 Department of Plant and Microbial Biology, University of California, Berkeley, CA USA

**Keywords:** Gene expression, Chromatin analysis, High-throughput screening, Plant molecular biology

## Abstract

Progress in sequencing, microfluidics, and analysis strategies has revolutionized the granularity at which multicellular organisms can be studied. In particular, single-cell transcriptomics has led to fundamental new insights into animal biology, such as the discovery of new cell types and cell type-specific disease processes. However, the application of single-cell approaches to plants, fungi, algae, or bacteria (environmental organisms) has been far more limited, largely due to the challenges posed by polysaccharide walls surrounding these species’ cells. In this perspective, we discuss opportunities afforded by single-cell technologies for energy and environmental science and grand challenges that must be tackled to apply these approaches to plants, fungi and algae. We highlight the need to develop better and more comprehensive single-cell technologies, analysis and visualization tools, and tissue preparation methods. We advocate for the creation of a centralized, open-access database to house plant single-cell data. Finally, we consider how such efforts should balance the need for deep characterization of select model species while still capturing the diversity in the plant kingdom. Investments into the development of methods, their application to relevant species, and the creation of resources to support data dissemination will enable groundbreaking insights to propel energy and environmental science forward.

## Abstract (web summary)

In this Perspective, Diane Dickel and colleagues review recent progress and opportunities in applying single-cell sequencing and microfluidics methods to plants. The authors highlight the need for new tools developed with plants in mind, and advocate for the creation of a centralized, open-access database to house plant single-cell data.

## Introduction

Biomass derived from the growth and harvest of plant feedstocks is a renewable and sustainable resource for the production of energy and materials. The global energy supply increasingly relies on robust and scalable bioenergy resources, which contribute to both energy security and the sustainability of energy production. Likewise, biomaterials derived from plants, algae, and microorganisms are growing in importance for a breadth of applications. Currently, available plant feedstocks require substantial amounts of land, water, and mineral resources, and their associated agricultural practices have considerable environmental impacts. To develop a more sustainable bioenergy and biomaterials portfolio for the future, we must significantly advance our understanding of how feedstock crops can be improved to tolerate and thrive in a continuously changing environment.

Critical to this understanding is knowing how the genome of a plant or other environmental organism (e.g., plant-associated bacteria or fungi) contributes to productivity, as this will empower breeding and bioengineering programs to enhance bioenergy and biomaterial production. The genomics era has significantly contributed to this cause by inspiring major investments into exploring the mechanisms underlying complex biological processes, principally through the use of global profiling strategies to measure RNA (transcriptomics), protein (proteomics), or metabolite (metabolomics) levels in plants. For example, recent work has leveraged multiple global profiling tools, such as genome-wide association surveys, transcriptomics, and proteomics to better understand how sorghum, an important bioenergy crop, responds to drought^1,2^. This work uncovered new hypotheses regarding how photosynthesis and the soil environment influence drought tolerance. While these powerful methods have already revealed major insights into the biology of bioenergy feedstock plants, they have been limited to surveys of whole organisms or complex tissues. Plant tissues consist of numerous distinct cell types, each with a specialized function within the context of that tissue or organism. Thus, each cell type will likely exhibit different molecular behaviors in response to an environmental challenge or produce a unique combination of metabolites or other products^3^. However, signals associated with specific cell types are averaged with, and thus diluted by, all of the other cell types present in the sample when profiling whole tissues using conventional bulk methods. Therefore, there is a need to develop molecular profiling methods that can evaluate individual cells or cell types for a more accurate understanding of how plant feedstocks can maintain productivity under environmental stress or design more rational plant engineering strategies for the sustainable generation of bioproducts.

Recently, there has been an explosion in methods that profile global biomolecule expression patterns in individual cells derived from complex tissues, which has revolutionized the way we can study and think about biological organization^4^. A major goal of these single-cell characterization methods is to divide cells from tissues into discrete classes (cell types or states), identify a unique transcriptional profile for each cell type, associate these with specific cell type functions, and define how cell types relate to one another functionally or developmentally (i.e., early, versus late developmental stages). Once the full complement of cell types is well defined, computational methods can be employed to address a wide range of questions, such as: what each cell type produces and how cell types respond to a variety of perturbations (e.g., environmental conditions or genetic mutation). These types of analyses have great potential to yield a more complete understanding of the function of cell populations, their adaptive and plastic properties, and sophisticated molecular toolboxes for biotechnological engineerings, such as regulatory sequences that can activate a gene or pathway within a particular cell type after exposure to a specific stimulus. Thus, single-cell characterization technologies comprise a powerful new suite of methods to study biological heterogeneity and promise to deliver a much deeper understanding of how organisms function as a unified collection of cell types.

In this perspective, we discuss the successes, challenges, and potential of single-cell molecular profiling methods in plant biology. First, we briefly introduce the current state of single-cell technologies and their use thus far on plant species. Next, we highlight major environmental and bioenergy research areas that would be particularly enhanced by the use of these methods. We address challenges that must be overcome for the wider adoption of these techniques for plants and other environmental organisms. Finally, as the plant biology field moves forward to build community-wide data and other resources to support single-cell biology, we discuss characteristics of such efforts that would facilitate environmental and energy biology.

## The technologies

### Single-cell transcriptomics methods

The most widely used of these new technologies, single-cell RNA sequencing (scRNA-seq), works by using microfluidics and barcoded DNA particles to capture whole transcriptomes of single cells^5^. Cutting-edge scRNA-seq methods can capture expression for tens of thousands of cells in a single experiment^6^. While microfluidics-based methods have the power to profile cell populations en masse, any spatial information (how those cells were organized within the larger tissue) is lost because the tissues must be first dissociated into individual cells for profiling. Newer sequence-based imaging methods (e.g., Slide-seq^7^, HDST^8^, Visium^9^, merFISH^10^, FISSEQ^11^, Nanostring^12^) hold great promise to impart spatial information to transcriptomic data. Some of these methods^7,9^ work by arraying barcoded particles along a 2-dimensional surface, then exposing this array to a thin tissue section to capture spatially resolved transcriptomes of individual cells or even subcellular compartments. Others use fluorescently labeled oligonucleotide mixtures that can be manipulated to report the position of hundreds to thousands of transcripts in a single specimen. These methods have been applied to a rapidly growing number of animal tissues, genotypes, and species to build extremely high-resolution profiles of gene expression. They have also been used to uncover novel cell types, infer gene regulatory networks, and understand how developmental processes unfold within highly heterogeneous biological specimens.

### Recent progress in plant single-cell “omics”

**Fig. 1:**
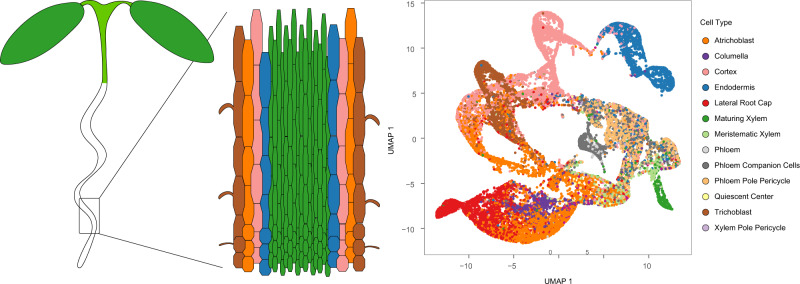
scRNA-seq of Arabidopsis root. Root development has recently been extensively characterized at the single-cell level in a series of scRNA-seq studies of root cell protoplasts. These methods can confidently identify all major cell types within roots and can begin to shed light on developmental trajectories that underlie root growth. Left, cartoon of generic plant root with different cell layers colored by their major cell type. Right, schematic of single-cell transcriptome data from plant roots.

While single-cell transcriptomics is now routinely and widely used in animal research programs, it has yet to be firmly rooted in plant or fungal research communities, which limits the ability to leverage this powerful set of tools to address current bioenergy and environmental challenges. Very recently, a number of groups independently addressed this technological gap by performing the first set of scRNA-seq studies on Arabidopsis root cells^13–18^. These studies identified nearly all major expected cell types, and many identified subclasses of cell types that were not previously well defined. Furthermore, these studies were useful in (1) characterizing complex signaling networks important for root development; (2) identifying a biphasic switch essential for xylem cell development^19^; (3) detailing the developmental progression of the endodermis^13^ and hair cells^17^ of the Arabidopsis root, and (4) profiling the initiation and development of lateral roots^18^. These datasets are being integrated together to form a comprehensive map of plant roots at an unprecedented level of detail (Fig. 1)^20^. Apart from plant roots, there is growing interest in using scRNA-seq technologies to profile the development of other important plant tissues, including leaf^21,22^, flower^23^, pollen and sperm^24^, and seed endosperm^25^. Single-cell RNA-seq has also moved beyond Arabidopsis, with studies emerging for tomato^26^, rice^27^, maize^28–31^, and moss^32^. Beyond single-cell RNA-seq, epigenomic profiling afforded by single-cell ATAC-seq has become increasingly used in plants^33–35^ and is ideally suited to explore gene regulation, elucidate regulatory networks, and even more finely classify cell types. scRNA-seq and scATAC-seq datasets are highly complementary and can be combined effectively^34^. Despite their demonstrated utility in mammalian systems, single-nucleus bisulfite sequencing^36^ (snmC-seq) and chromatin immunoprecipitation sequencing^37,38^ (scChIP-seq) have not yet been demonstrated in plant species, possibly due to the high cost and coverage requirements and relatively low throughput, though this would provide valuable information for how DNA modifications and chromatin influence cell behavior. Apart from the microfluidics-based technologies, spatial transcriptomics^39,40^ methods have also begun to be applied to plants, including Arabidopsis, poplar, and spruce. These methods match gene expression to specific physical locations within organisms but require a non-trivial amount of optimization, and thus are still in their infancy in plants. Finally, single-cell proteomics is also making advances, though this technology is still nascent and not widely used in either animals or plants^41,42^.

Despite significant recent progress, the plant community still lags far behind the animal field with respect to the adoption and application of single-cell methodologies and, with some exceptions^20,43^, computational tool development. While the animal field has leveraged single-cell methods to perform massive combinatorial screens^44,45^, study disease heterogeneity^46^, and whole-organism^4,47^ and cross-species^48^ profiling efforts, in plants applications have been limited to studying developmental processes associated with individual tissues^13–17,21^ and a limited number of treatment/control experiments^13,15,27^. This is due in part to some of the technical limitations described in more detail below (see the section “Critical technological and analysis challenges”).

### Future technology goals

Building upon initial profiling efforts to understand previously uncharacterized cell types, tissues, and species will prove critical in the near future if we want a better understanding of how individual cells behave in stressful environmental conditions, how plants interact with their microbiota, or how to better engineer plants or fungi for efficient and sustainable bioproduct synthesis. In addition, it is unclear how single-cell profiling will be applied to the non-model plant or fungal species, but such an expansion would have enormous benefits for biotechnology applications. While in principle, single-cell methods can be applied to any organism with a sequenced and annotated genome, in practice, a more universal method for cell or nucleus isolation and processing is needed to democratize the technology. Single-cell profiling could also significantly enhance our ability to annotate gene function in non-model plant species. Moving beyond transcriptomics and epigenomics, there is a rising need to describe complex states of individual cells, with a particular emphasis on elucidating metabolic pathways. Along these lines, nascent single-cell metabolomics, proteomics, and imaging technologies show great promise in helping to address this unmet need. One could potentially envision integrated workflows in the future that combine multiple technologies on a single device to monitor several features from the same cell. There has also been recent progress in developing mass spectrometry-based metabolite imaging for spatially profiling metabolite quantities in plants^49^. Further development of this technology will nicely complement advances in other single-cell methods.

## Grand research challenges for single-cell profiling of plants

As the technological capabilities to profile plants at the single-cell level improves, we believe that three specific areas, in particular, should be of immediate interest. These include a detailed understanding of how plants respond to biotic and abiotic environmental factors, opportunities for improved functional annotation of genomes, and applications for the production of bioproducts and biomaterials. To support these goals, technological and analytical challenges must be overcome, which will require significant investment, including a centralized resource to facilitate the sharing of single-cell data and identification of potential funding avenues for single-cell science in energy and the environment.

### Plant responses to biotic and abiotic interactions

**Fig. 2:**
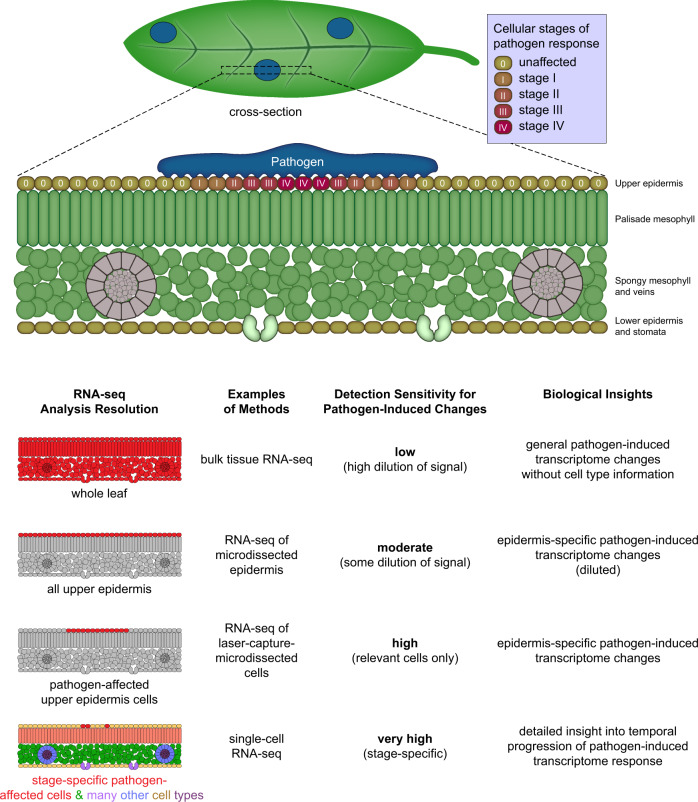
Advantages of using single-cell RNA-seq to study plant-pathogen interactions. Relatively few plant cells interact directly with most pathogens. However, these local interactions often determine disease severity. Thus, understanding gene expression in these few cells could be valuable for enhancing resistance. Unfortunately, bulk tissue RNA-seq greatly dilutes the signal from interacting cells, and signals from genes upregulated throughout the leaf in response to pathogens can mask expression changes in the interacting cells. While methods like microdissection can improve the signal-to-noise ratio to a degree, they are labor-intensive and not universally applicable to all pathogens. Thus, the increased cellular resolution promised by single-cell RNA-seq could revolutionize our understanding of plant-microbe interactions.

Emerging single-cell technologies are expected to enable impactful discoveries in studies of plant responses to their environment. Examples of interactions with particularly high relevance include pathogenic infections and mutualistic associations with nitrogen-fixing bacteria, as well as abiotic environmental conditions such as drought, heat, or limited nutrient availability. Both pathogenic and commensal microorganisms typically interact with very specific subpopulations of cells in a host plant, with many (or most) plant cells not in direct contact with or infected by specific microbes. For example, arbuscular mycorrhizal fungi specifically target only a subset of cortical cells of the plant root. Current methods of performing RNA-seq on bulk tissue or cell populations isolated by fluorescence-activated cell sorting (FACS) of reporter-labeled plant lines massively dilute any signal originating from affected cells in the plant (Fig. 2). Microfluidic-based single-cell RNA-seq, in combination with emerging spatial transcriptomics methods, holds great promise for elucidating cell-specific responses to pathogenic infections or other perturbations. Of particular importance for this research area is the development of methods that are capable of capturing RNA molecules from both eukaryotic and prokaryotic organisms in the same experiment, since current methods are limited to eukaryotic cells that have mRNA polyadenylation. Drought is another high-priority focus area in this domain. In addition to the long-term goal of understanding the biological effects of decreased water availability caused by changing environments, single-cell methods could be used in the short term to better understand which experimental systems that are currently used to simulate drought in the lab are the most biologically relevant. With resource investments in developing tissue preparation methods and new technologies, along with the study of targeted scientific questions, single-cell technologies have the potential to revolutionize plant environmental science.

### Better annotation of plant/fungal/algal gene function

**Fig. 3:**
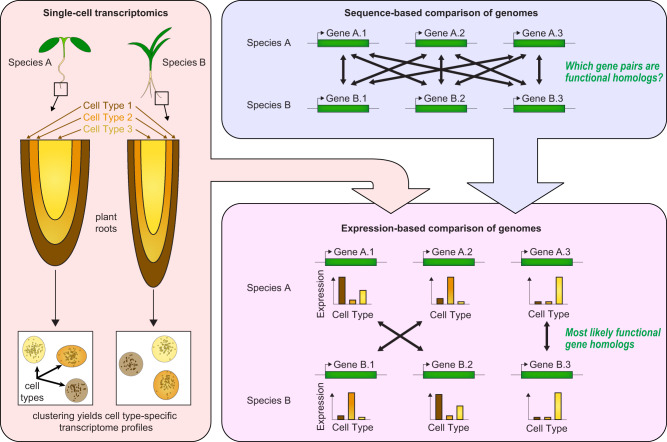
Using single-cell transcriptome data to improve the comparative annotation of plant genomes. Expression profiles across multiple cell types derived from single-cell transcriptome data of tissues from different plant species (left), in combination with sequence homology-based comparison of protein sequences (top right), can be used to identify functionally homologous genes across different plant species (bottom right), thereby substantially enhancing the ability to assign functional knowledge from deeply annotated model species correctly to other species that are of interest to bioenergy and biomaterial production.

A second major scientific focus area where single-cell technologies could have a substantial impact is in the functional annotation of genes from plants, fungi, and algae. For example, DOE Joint Genome Institute portals (Phytozome, MycoCosm, PhycoCosm) host the sequences of >180 plant genomes (from >100 distinct species), along with >1600 fungal and >50 algal genomes. Newly sequenced and assembled genomes are run through standardized annotation pipelines, which include using DNA sequence homology to genes in well-studied model species (e.g., Arabidopsis for plants) to infer the function of genes from the newly sequenced species. However, due to the ubiquity of large gene families with similar sequences in plants, identification of exactly homologous gene pairs between species is often challenging. Further complicating this challenge, functional understanding for most genes, even in well-studied species, is lacking. This can be mitigated by the use of gene expression information, in addition to sequence homology. RNA-seq data derived from different bulk plant tissues is already being used to define “expressologs”, which are pairs of genes with similar expression profiles across general tissues in the species being compared. With scRNA-seq data, it will be possible to perform such analyses across dozens of cell types, thereby increasing the accuracy of the resulting annotations and inferred gene functions (Fig. 3). Indeed, a recent study leveraging Arabidopsis single-cell data found extreme cell type-specific expression bias among pairs of homologous genes (gene duplicates)^50^. Using this information could result in substantial improvements to plant functional gene annotations across species.

Beyond using quantitative expression information for identification of functional gene homologs between species, nascent technologies for capturing full-length transcripts from single cells (e.g., scIso-seq^51^ or Smart-seq3^52^) also have the potential to identify cell type-specific mRNA isoforms, adding another important layer of functional genomic annotation to transcriptome data. Generation of scRNA-seq and/or spatially resolved transcriptomics data for tissues from a panel of species would serve as a starting point to build analysis tools and assess their utility. The initial panel should include a diverse set of species, including both better studied models (e.g., *Arabidopsis thaliana*, *Brachypodium distachyon*) and additional species selected for phenotypic or phylogenetic diversity, which would maximize the potential as a general resource. Alternatively, the initial panel might be selected based on more pragmatic criteria, like the availability of tissue preparation methods. Once established, this program could then be scaled to include a much wider diversity of species. Development of better cell culture transformation systems for environmental species would complement this effort. Such methods will be essential for performing high-throughput gene functional characterization in environmental species using Perturb-seq^44^ screens, or conceptually similar methods such as CROP-seq^53^ or CRISP-seq^45^. Perturb-seq, which has been used extensively in cultured human cell lines, combines CRISPR/Cas9-mediated gene knockout with scRNA-seq to elucidate gene regulatory networks, and it could be harnessed to study the importance of different genes and pathways under altered growth conditions, for example in the absence of specific nutrients. These methods could potentially be adapted to plants using a source of relatively homogeneous cells (e.g., leaf mesophyll protoplasts, or protoplasts derived from callus tissue). While these cells may behave differently than they would *in planta*, the high-throughput gene expression manipulation afforded by Perturb-seq and related methods would greatly accelerate gene function prediction and serve as a powerful hypothesis generation tool. Collectively, single-cell technologies performed on a diverse panel of plant species and tissues, along with the application of high-throughput functional screens, could substantially improve our understanding of gene function.

### Improving production of bioproducts and biomaterials

**Fig. 4:**
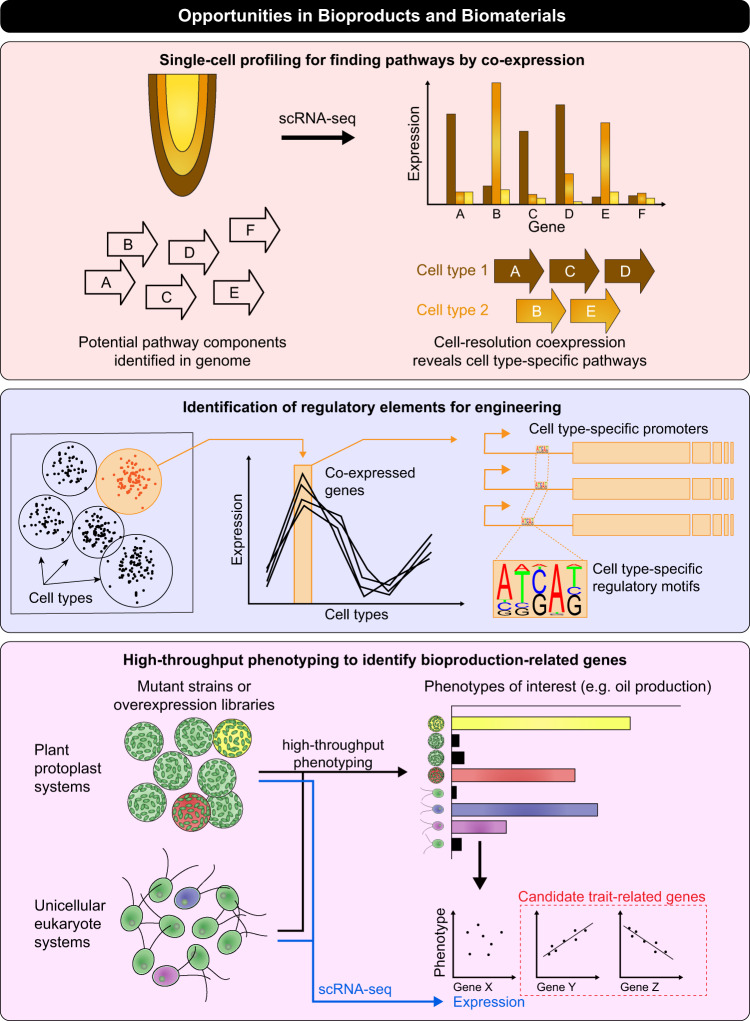
Using single-cell methods in bioproducts and biomaterials applications. Top panel: single-cell resolution data can be used to find genes in biosynthesis pathways by identifying co-expressed genes in individual cells or cell types. Middle panel: single-cell expression data can identify cell-specific and condition-specific building blocks, as genes that co-vary across clusters of cells are likely regulated by common components (e.g., transcription factors). This can be exploited to identify promoters useful for bioengineering applications where production in a specific cell type is desired. Bottom panel: improvements to bioproduction targets in plant or algal systems could be achieved through correlating high-throughput phenotyping and single-cell resolution “omics” data. High-throughput analyses of mutant strains or libraries containing engineered biosynthetic clusters could be used to identify or verify which genes and pathways are necessary for the production of specific products and to optimize for higher production yield.

In addition to elucidating a foundational understanding of metabolism in plants and microbes, single-cell data will be important for both discovering natural product pathways and for successfully leveraging genome engineering and synthetic biology methods to produce biomaterials efficiently. Single-cell techniques could aid in predicting and refactoring biosynthetic pathways, optimizing bioproduction, and generating predictive metabolic models. One important application for single-cell technologies will be in the area of biosynthetic pathway discovery. Some bioproducts produced by plants are synthesized predominantly in one or a few specific cell types (e.g., suberin in root endodermis cells), and biosynthetic pathways are known for only a small subset of plant products. While many types of enzymes can be predicted from genome information based on sequence similarity to related proteins, this information generally is insufficient to understand which genes work as part of a common pathway in vivo. For example, sequence similarity often enables robust prediction of enzyme class, such as “hydrolase” or “reductase”, but rarely predicts the substrate(s)^54^. High-throughput single-cell metabolomics and proteomics methods would be invaluable for systematically mapping where naturally occurring bioproducts are produced in plant tissues. For those products restricted to specific cellular populations, cell type-specific expression profiling could be used to narrow down components of a common biochemical pathway by identifying sets of enzymatic genes that are co-expressed in the same cell type (Fig. 4, top). Additionally, single-cell expression information has the potential to improve bioengineering processes. Specific cell types are likely to provide better host environments than others for bioproduction because of the availability of substrates/cofactors, the absence of inhibitors, or resistance to product toxicity. Single-cell technologies applied to diverse plant tissue types are widely expected to aid identification of cell types that are best for making a product. More importantly, these approaches can also identify promoters or other regulatory elements that can direct expression to those cell types with high specificity, thereby providing crucial building blocks for biosynthetic engineering (Fig. 4, middle). Finally, single-cell transcriptome profiling can be coupled with single-cell proteomics, antibody labeling or high-throughput microfluidic phenotyping systems using plant protoplasts or unicellular eukaryotes, such as algae. This approach could be used to assess, for example, libraries of cells engineered to overexpress candidate genes/pathways or saturation mutagenesis libraries (Fig. 4, bottom). By combining single-cell gene expression and phenotyping information, it will be possible to correlate transcript abundance with cellular measurements, enabling a rapid assessment of thousands of genetic manipulations for their phenotypic impact. Example applications for this approach include the search for genes and pathways that increase production of a biomaterial of interest in a given species.

Adapting single-cell technologies (transcriptome, proteome, and metabolome) to fungal and algal species, in addition to plant cell suspension systems, will be particularly important for improving bioproduct and biomaterial production. These methods could additionally provide a foundational understanding of culture population diversity, facilitate pathway optimization through parallelization, elucidate synthesis dynamics, and reveal whether heterogeneous populations are important for synthesis. For instance, the synthesis of some bioproducts may require a combination of cell types and a mechanism for transport of metabolites between cell types. The development of methods that allow sampling of multiple different molecule types in parallel (e.g., mRNA AND metabolites) or imaging in combination with molecular profiling, along with sample preparation methods that do not substantially alter cellular phenotype for a diversity of commonly used production species/strains, will be critical for bioproduct synthesis applications.

## Critical technological and analysis challenges

### Tissue preparation

Plant, algal, and fungal species, in contrast to animals, have complex polysaccharide cell walls that must be removed or permeabilized for single-cell characterization. This challenge has substantially hindered the application of these methods to such species. Methods of using enzyme cocktails to remove cell walls (i.e., protoplasting) are available for some species and tissues (e.g., Arabidopsis root), but cell wall composition differs from species to species and even between tissues of the same plant, so these methods are not universally applicable. Additionally, these dissociation/permeabilization methods impart unintended transcriptional or metabolic changes to the cells that, combined with the enormous variation in size of plant cells between species, organs, and tissues, may preclude the ability of some cell types to be universally and accurately profiled by many of the microfluidics-based single-cell technologies commonly applied to mammalian cells. However, the benefits of successful protoplast isolation are that the whole cellular complement of biomolecules can be potentially sampled, which may prove important, especially for low-abundance transcripts or quantification of proteins that are outside of the nucleus. Isolation of nuclei, rather than whole cells, and cellular fixation methods (e.g., methanol treatment), are attractive alternatives for single-cell science in plants and fungi. However, there would be great value in revisiting and reviving historical methods of cell isolation and tissue preparation^55–59^. Overall, developing better tissue, cellular, and nuclear preparation methods for plants, fungi, and algae is an immediate focus area that would broadly enable the application of single-cell methods to environmental and energy science.

### New single-cell and spatial technologies

One of the more exciting new areas in single-cell characterization is the development of technologies beyond the commonly used microfluidic scRNA-seq methods. One limitation of the microfluidic methods is that they require tissue dissociation, and spatial information about where a specific cell came from within the tissue is lost. Several such methods (e.g., Slide-seq^7^), while not currently single cell-resolution, provide spatially resolved gene expression for tissue slices. Additionally, methods such as MERFISH^10,60^ can reveal gene expression down to specific sub-regions within cells but currently require substantial investments and specialized expertise in microscopy equipment. There is a great need for the development of high-throughput single-cell transcriptomics methods that could capture information for both eukaryotic and non-eukaryotic organisms at the same time since current widely used methods are restricted to reading RNA transcripts that have polyadenylation signals. This prohibits their use for profiling bacteria or archaea that are interacting with plants, an essential element for fully characterizing complex soil communities. One potential solution lies in adapting the chemistry of single-cell reagents to not rely on pre-existing polyA sequences, as has been recently demonstrated for high-throughput plate-based barcoding assays of bacteria^61,62^. If this translates well to spatial transcriptomics assays, it could also solve the more difficult challenge of how to quantify the transcript abundance of both plant and prokaryotic cells in a symbiotic system while preserving their spatial context. The study of plant/microbial interaction systems poses the additional challenge of spatial complexity in three dimensions. For instance, bacteria are often non-uniformly distributed across multiple planes when colonizing plants. Recently, methods incorporating polyadenylation enzymes that target mRNA from bacteria have been demonstrated to overcome limitations in prokaryotic transcriptome capture^62,63^. Still, other technologies have shown the possibility of describing transcriptomic changes in 3-dimensional space (e.g., FISSEQ^64^ and STARmap^65^), while computational methods are being developed to accurately segment 3-dimensional images of plant tissues into their composite cells for detailed analysis^66^. Further application and integration of such methods would substantially benefit the study of plant-microbe interactions in the environment. Currently, many single-cell or spatially resolved transcriptomics methods result in data that is restricted to specific regions of genes (e.g., the 3′ end of transcripts for droplet-based scRNA-seq), and sequencing full-length transcripts would provide useful information about gene isoforms, which may be necessary to address some questions as a complement to the current, higher-throughput methods. Beyond gene expression, there is a strong need for high-throughput single-cell proteomics and metabolomics methods. Such methods are in development but have throughputs that currently lag substantially behind transcriptomics methods or require specialized antibodies, limiting their application to specific panels of proteins^67,68^. Emerging methods such as CITE-seq^69^ combine scRNA-seq with antibody labeling to interrogate gene expression and the repertoire of cell surface proteins for individual cells in the same experiment. In addition to single-cell profiling methods, there is a need for better methods to validate single-cell results, including improved in situ hybridization protocols such as single-molecule FISH^70^ for plants, as well as faster and more efficient ways to generate reporter lines. Additionally, the application of technologies like the 10x Genomics Visium platform could also serve as a powerful validation and discovery tool^39^. With the exception of an early, low resolution incarnation of spatial transcriptomics used to profile plant shoot tissue^39,40^, most of these exciting new technologies have been exclusively applied to animal systems. However, there is great promise that more modalities of biomolecule profiling will soon advance our understanding of plants.

### Analysis methods

Complementary to novel microfluidics methods and advanced molecular biology reagents and protocols, innovative computational methods employing statistical tools rooted in machine learning have been the third technology pillar that has enabled breakthrough advances in single-cell approaches in recent years. Examples of these approaches include computational strategies to capture “free” information from existing data, including developmental trajectories (e.g., Monocle^71^, Palantir^72^, SlingShot^73^, and CellRank^74^), RNA dynamics (e.g., RNA velocity^75^ and scVelo^76^), methods to integrate disparate and multimodal datasets (e.g., Seurat^77^, Harmony^78^, Symphony^79^), methods that implement differential gene expression analysis (e.g., MILO^80^), and several end-to-end pipelines that implement large collections of tools in a single computational ecosystem (e.g., Seurat^81^, Monocle^71^, scanpy^82^). While many of the tools already developed for analysis of single-cell data from mammalian tissues will be applicable to analysis of plant data sets, and indeed some tools were specifically developed for the analysis of plant single-cell data (e.g., Asc-Seurat^43^, COPILOT^20^, Socrates^33^), several computational challenges remain that are unique or of particular consideration to plants and would benefit from the development of still more new tools and databases. For example, as research moves from Arabidopsis roots in the first wave of studies to new species and tissues, how do we know if cell clusters represent true cell types if no high-quality cell type-specific markers are already known? Furthermore, how will typical mapping pipelines for scRNA-seq perform when aligning to transcripts from poorly annotated genomes? To enable robust and valid data analysis, we will need to adapt or develop tools for cross-species comparison that do not require a one-to-one gene mapping between organisms^83^. In considering the funding landscape for such efforts with a focus on species relevant to bioenergy applications, DOE, with its strong history of driving plant and microbial computational tool development, would be particularly well-positioned to support such efforts.

## Data resources to enable environmental science

### A pressing need for an open plant cell atlas data resource

In order to be useful to the broader plant science community, a publicly accessible platform that conforms to the FAIR (Findable, Accessible, Interoperable, and Reusable) data principles (https://www.go-fair.org/fair-principles/) for sharing results is urgently needed. Large-scale consortia in the human biology field, such as the Human Cell Atlas and the Encyclopedia of DNA Elements (ENCODE), have made routine the immediate and open sharing of data, often even in advance of publication. In contrast, research communities that have organized around different plant species often have vastly different data-sharing practices, and journal publication requirements alone have proven inadequate to compel consistent data sharing. A vision is currently emerging for a Plant Cell Atlas^84^ that would include not only single-cell transcriptomics data but multi-scale imaging, proteomics, and other data types, as well. A unified portal for single-cell and other data from the plant community would greatly facilitate the widespread movement toward FAIR data principles. There was universal agreement that such a platform, like the Human Cell Atlas, should have international support and accessibility and not be wholly funded by a single country or funding agency. In addition to enabling data sharing, such a resource would have the added benefit of establishing high standards for data quality, enable consistency in data analysis, and provide innovative and powerful data visualization tools. Having a unified platform supporting multiple plant research communities would facilitate solutions to emerging problems, such as how to define analogous cell types between different species. It could also serve as a platform to support the sharing of information beyond results, such as tissue dissociation or preparation protocols.

### Deep or wide?

**Fig. 5:**
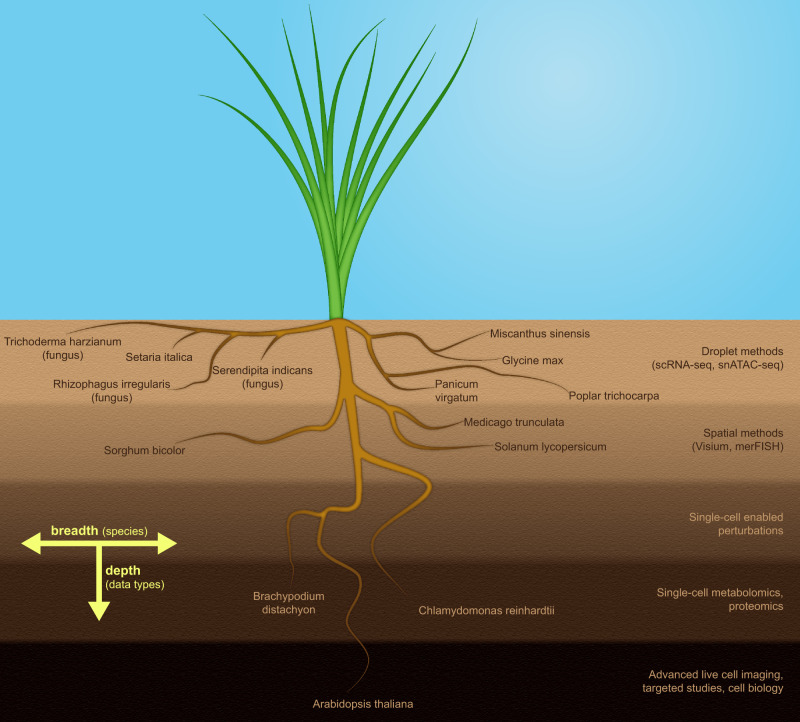
Deep or wide?. The schematic root system of a hypothetical plant, covering a large area close to the surface while also penetrating deeper soil layers with some of its roots, provides a visual metaphor for the need to complement “wide and shallow” characterization of many species using a select subset of single-cell assays with “deep and narrow” in-depth studies of select model species using the full arsenal of single-cell methods available.

An overarching question is whether the effort to establish a Plant Cell Atlas should focus exclusively on very deep characterization of a single plant species or generation and curation of data from a wide variety of species. Given the existing research and database infrastructure, a single-species effort would almost certainly focus on *A. thaliana*. Pragmatically, it makes inherent sense if resources are limited to commit to completing a deep, multimodal characterization of a single species. Such a dataset would have the best potential for being able to integrate different types of data with machine learning and similar strategies to construct accurate systems-level models of an entire plant. However, this “narrow- and-deep” approach has inherent limitations for understanding aspects of plant biology that Arabidopsis either does not perform or poorly models, such as a C4-mode of carbon fixation or many of the general anatomical, physiological, and molecular features that facilitate high-yield biomass production in bioenergy grasses. A “wide-and-shallow” effort to perform a subset of the proposed molecular profiling, such as only single-cell or spatial transcriptomics, on tissues from a larger panel of phylogenetically or phenotypically diverse plant species would be highly complementary to a deep characterization of Arabidopsis (Fig. 5). This approach would provide critical baseline information about a variety of plant species important for the environment, energy, biosynthesis, and food production. It would establish a centralized open data resource for the research communities working on these species and inform downstream experimental studies, genome annotation, and genetic engineering of these organisms. Ideally, both wide and deep efforts would not be mutually exclusive and would work together to coordinate data production and release through a centralized portal that would broadly serve the plant biology community.

## Summary and conclusions

Over the past several years, an astounding array of new single-cell technologies has driven unprecedented advances in the biomedical sciences. New methods that leverage advanced experimental and computational tools provide single-cell resolution transcriptome and epigenome information and are complemented by nascent methods for proteome, metabolome, and spatially resolved transcriptomics at the single-cell level. Within the past few years, we have begun to see some of these same approaches demonstrated in plants, fungi, and algae. While significant technical challenges still need to be overcome before these techniques can be broadly applied to the wide array of species that are of interest to energy and environmental studies, this initial wave of published studies is only a harbinger of the powerful discovery opportunities these methods will enable. Thus, the time is ripe for focused investments into the development and adoption of single-cell methods to drive the next wave of biological innovation for energy and environmental science.

Single-cell molecular profiling methods are expected to have the same paradigm-shifting potential for plant and environmental biology as they have already had in the biomedical sciences. In plant science, cell type resolution has always been ‘the holy grail,’ and single-cell methods are expected to provide a direct window into multiple areas of plant biology (Box 1). Applying single cell methods to microbial and fungal species, in addition to plants, would enable greater understanding of how plants and microbes interact in commensal, competitive, and pathogenic relationships. In addition, fundamental insights into cell state properties of eukaryotic microbes could be used to improve bioreactor-based production. Lastly, single-cell measurements of individuals across a population can capture properties such as life cycle, measure population heterogeneity, distinguish between stochastic and regulated processes, and guide how desired cell states can be selected through engineering. This new frontier for single-cell science is likely to face unique challenges, but these issues could be addressed through targeted investments in technology development and a data-sharing platform. Advances in single-cell technologies will have exciting and far-reaching impacts when widely applied to plants, fungi, and microbes, and will be transformative for both our understanding of environmental biology and for trait engineering for bioenergy and biomaterials.

Box 1. Probing the actions of individual cells within a population has enormous potential to reveal fascinating biological details. These include

- *Insight into plant cell type function*: Nearly all biological functions a plant executes in vivo occur through the interplay of many different cell types with highly specialized functional profiles. Resolving the molecular blueprint (transcriptome, proteome, metabolome, etc.) of specific cell types across plant tissues will give direct insight into how cells perform their respective specialized roles.
- *Insight into plant development*: Understanding the development of plants is critical for improving traits such as biomass yield. Advanced single-cell transcriptome analysis strategies, such as “pseudotime” and “RNA velocity”, enable unprecedented insight into developmental trajectories of cell types during plant development.
- *Understanding plant responses to environmental factors*: Many factors affecting the response of plants to environmental factors, such as pathogens, drought, nutrients, climate, or soil are likely driven by very specific processes taking place only in subsets of their cell types. Single-cell methods will make it possible to deconvolute these responses and assign specific aspects of the organismal response to the cell types they occur in.
- *Functional annotation of plant genes and gene families*: Plants tend to have large gene families and complex, polyploid genomes, which creates major challenges in correctly identifying functional gene homologs across related plant species. Single-cell technologies provide high-resolution gene expression data that can be used to enable correct assignment of functional orthologs across species, making it possible to correctly extrapolate gene function from deeply annotated model species to crops of interest. Methods combining single cell technologies with high-throughput genome engineering could elucidate the function of genes that have not been previously characterized.
- *Identification of targets for bioenergy crop improvement*: As scientists and breeders strive for predictive engineering of plant traits, a detailed understanding of how gene networks are composed and regulated in response to environmental input at a cell type level will substantially accelerate progress towards the creation of more productive and sustainable energy crops.
